# Altered HIV-1 mRNA Splicing Due to Drug-Resistance-Associated Mutations in Exon 2/2b

**DOI:** 10.3390/ijms23010156

**Published:** 2021-12-23

**Authors:** Lisa Müller, Wiebke Moskorz, Anna-Lena Brillen, Frank Hillebrand, Philipp Niklas Ostermann, Niklas Kiel, Lara Walotka, Johannes Ptok, Jörg Timm, Nadine Lübke, Heiner Schaal

**Affiliations:** Institute of Virology, Medical Faculty, Heinrich Heine University Düsseldorf, 40225 Düsseldorf, Germany; Wiebke.Moskorz@med.uni-duesseldorf.de (W.M.); AnnaBrillen@outlook.de (A.-L.B.); frank.hillebrand@outlook.com (F.H.); Philipp.Ostermann@uni-duesseldorf.de (P.N.O.); Niklas.Kiel@hhu.de (N.K.); Lara.Walotka@hhu.de (L.W.); johannes.ptok@uni-duesseldorf.de (J.P.); Joerg.Timm@med.uni-duesseldorf.de (J.T.); Nadine.Luebke@med.uni-duesseldorf.de (N.L.); schaal@uni-duesseldorf.de (H.S.)

**Keywords:** splicing, HIV-1, SREs

## Abstract

The underlying molecular mechanism and their general effect on the replication capacity of HIV 1 drug-resistance-associated mutations is often poorly understood. To elucidate the effect of two such mutations located in a region with a high density of spicing regulatory elements on the HIV-1-splicing outcome, bioinformatic predictions were combined with transfection and infection experiments. Results show that the previously described R263K drug-resistance-associated integrase mutation has additionally a severe effect on the ESE2b splicing regulatory element (SRE) in exon 2b, which causes loss of SD2b recognition. This was confirmed by an R263R silent mutation with a similar predicted effect on the exon 2b SRE. In contrast, a V260I mutation and its silent counterpart with a lower effect on ESS2b did not exhibit any differences in the splicing pattern. Since HIV-1 highly relies on a balanced splicing reaction, changes in the splicing outcome can contribute to changes in viral replication and might add to the effect of escape mutations toward antiviral drugs. Thus, a classification of mutations purely addressing proteins is insufficient.

## 1. Introduction

Human immunodeficiency virus 1 (HIV-1) infection is still not sterile curable to this day, although several promising results have at least demonstrated viral clearance from infected hosts in cell cultures and small animal models using genome-editing approaches [1]. Nevertheless, the development and current availability of antiviral treatment are already making significant contributions to improving quality of life and increasing life expectancy [2,3]. Country-specific studies and data from the World Health Organization and UNAIDS, respectively, suggest that overall, both the number of HIV-1 patients receiving highly active antiretroviral therapy (HAART) and the duration of treatment increased over the past years [4,5,6]. This, however, might contribute to increased incidents of drug resistance, especially in non-adherent patients or in areas with limited access to drugs and regular clinical monitoring [7,8].

On a molecular level, HIV-1 highly relies on host factors for replication, in particular the cellular splicing machinery [9]. Upon infection and reverse transcription of the viral RNA into the 9.7 kb proviral DNA, the assembled pre-integration complex (PIC) facilitates nuclear incorporation and subsequent integration of the HIV-1 genome into the host’s chromosome, which is then transcribed into a full-length precursor mRNA (pre-mRNA) [10,11]. For the production of the complete range of mRNAs needed for balanced expression of all viral proteins and functional replication, transcripts undergo extensive alternative splicing (AS), which yields approximately 50 different mRNAs species. Depending on their size, the resulting mRNAs are classified into full-length 9 kb, intron-containing 4 kb, and intron-less 2 kb mRNAs. Four major splice donor (SD) sites (SD1 to SD4), in combination with eight splice acceptor (SA) sites (SA1, SA2, SA3, SA4a,b,c, SA5, and SA7), contribute to the generation of the majority of mRNA isoforms, while the rare use of additional splice sites such as splice donor SD2b or splice acceptors SA4d or SA5b further enlarge the transcript repertoire [12,13,14,15,16,17].

In general, their recognition depends on the intrinsic strength of the splice sites, which can be scored by various algorithms such as the HBond score (HBS) [18] or MaxEnt scan [19]. A subset of HIV-1 3′ SA exhibit lower intrinsic strength scores, compared with cellular 3′ SA sites [20]; hence, they are likely more influenced by nearby splicing regulatory elements [21]. Additionally, the order of intrinsic splice site strengths does not correlate with the observed levels of mRNAs, which implies that *cis*-acting splicing regulatory elements add an additional layer of regulation and dominate splice site selection of HIV-1 mRNAs [22].

The compact HIV-1 genome contains numerous *cis*-acting regulatory RNA elements that can influence a variety of processes essential for viral replication including genomic RNA packaging, pre-mRNA processing, polyadenylation, and nuclear RNA export [17,23]. The sequence elements and changes thereof can subsequently influence the overall replication capacity of viruses [24]. In addition to altering the amino acid sequence, missense mutations but, indeed, also silent mutations in SREs, can affect viral replication by altering the binding of splicing regulatory proteins. This could then ultimately lead to altered splice site recognition and severely disrupt viral replication by altering the ratio of alternatively spliced mRNA isoforms required for effective replication [14]. Some of these Sanger or next-generation sequencing detected mutations exhibit the potential to alter the viral splicing patterns and thus change the viral proteome [25]. Such mutations might ultimately be associated with reduced sensitivity to antiviral agents.

Here, we analyzed two antiviral drug-resistance-associated mutations, V260I and R263K, located within the C-terminal domain of integrase. The HIV integrase is encoded in the *pol* gene and promotes the integration of the provirus into the host’s genome. The splice sites SA1, SD2, and SD2b are located within this distal region of *pol*. Among others, the splicing regulatory elements ESE-Vif, ESS2b, and ESE2b modulate their usage [17,26,27].

The aim of this study was to evaluate the influence of the V260I and R263K mutations on the alternative splicing of HIV-1 mRNA.

## 2. Results

**Figure 1:**
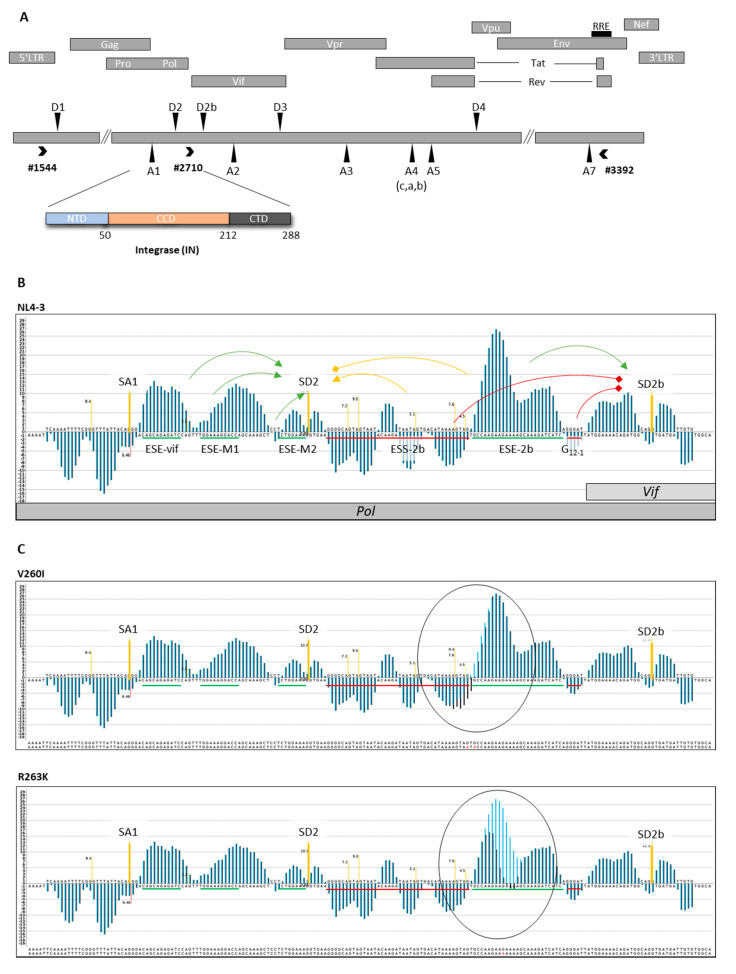
Organization and regulation of the HIV-1 genome: (**A**) the positions of the HIV-1 open reading frames are shown as grey boxes. Ribosomal frame-shifting allows the production of the viral enzymes protease (PR), reverse transcriptase (RT), and integrase (IN), which are also cleavage products of the Gag-Pol polyprotein. The 288 amino acids (32kDa) HIV-1 IN mediates the integration of the provirus into the host genome and contains three domains (i) N-terminal domain (NTD, AS 1-50), (ii) catalytic core domain (CCD, AS 50-212), and (iii) C-terminal domain (CTD, AS 212-288); (**B**) HEXplorer plot of the Integrase coding region is interspersed with well-characterized SREs that mediate the use of splice acceptor SA1 (MaxEnt 6.41) and both splice donors, SD2 (HBond score 10.7) and SD2b (HBond score 12.4). Positive regulation of splice sites (yellow bars) is marked in green, negative regulation in red, and intermediate regulation in orange. Positive areas in the HEXplorer indicate potential SR-protein binding, while negative areas indicate potential hnRNP protein binding. Parental sequences are depicted as black bars, while changes by mutations are depicted as blue bars; (**C**) impact of two secondary resistance-associated mutations on ESE2b depicted by HEXplorer plots. V260I (∆HZ_EI_ = −32.79), potentially slightly adding to the negative regulation of SD2b but increasing the enhancement of SD2. Mutation R263K (∆HZ_EI_ = −132.16) is likely to increase SD2 at the expense of SD2b.

In the C-terminal domain of the integrase coding region, i.e., the 3′ end of HIV-1 *pol*, both mutations, V260I and R263K, are located within splicing regulatory elements—namely, ESS2b and ESE2b (Figure 1A). The mutations were bioinformatically analyzed for their potential to impact splice site recognition of exon 2/2b by changes in the splicing regulatory elements. The bioinformatics tool used for the prediction, the HEXplorer tool [28], is one of various publicly available algorithms to analyze SREs and the consequences of mutations. It has already been employed to identify SREs within the HIV-1 genome [26,27]. The tool is based on hexamer frequencies in the exonic or intronic vicinity of splice sites where positive scores are based on 6-mers that are more frequently located upstream of splice donor sites, whereas negative scores indicate 6-mers that are more frequently located downstream of splice donor sites. The tool then evaluates sequence stretches in a sliding window according to the probability of sequence areas to act as SREs by posing as binding sites for splicing regulatory proteins, mainly of the SR or hnRNP family [28,29]. The consequences of nucleotide changes are given as differences in hexamer frequencies, calculated as ΔHZ_EI_. Thus, higher changes in the HZ_EI_ score indicate a higher potential to alter the binding potential of SREs. The two mutations analyzed in this study are of particular interest since HEXplorer evaluation suggested a certain potential to alter the splicing outcome. Compared with the parental sequence, the V260I mutation is predicted to have little effect on splice site recognition with a ΔHZ_EI_ of −37.79, whereas R263K is predicted to result in a more severe change in splicing outcome with a ΔHZ_EI_ of −132.16. Despite the two chosen mutations being located outside of the catalytic core of the Integrase, they are described as secondary resistance-associated mutations [30]. Additionally, R263K was recently described to decrease HIV integration and to be associated with therapy failure using Dolutegravir in HIV-1 treated patients [31,32,33].

The 3′ end of HIV-1 *pol* is of particular interest since it is interspersed with a dense network of splicing regulatory elements that have been shown to mediate the use of splice acceptor SA1 (MaxEnt 6.41) and splice donors SD2 (HBond score 10.7) and SD2b (HBond score 12.4) (Figure 1B) [22,26,34,35]. HEXplorer plots show the predicted impact of the two secondary resistance-associated mutations on the ESE2b SRE. V260I displays a change by an ∆HZ_EI_ of −32.79, suggesting a potential only minor addition to the negative regulation of SD2b and a slightly increased enhancement of SD2. Mutation R263K, however, largely alters the HEXplorer plot by an ∆HZ_EI_ of −132.16, and thus, this mutation is predicted to increase SD2 use while the support of SD2b should be drastically decreased (Figure 1C).

**Figure 2:**
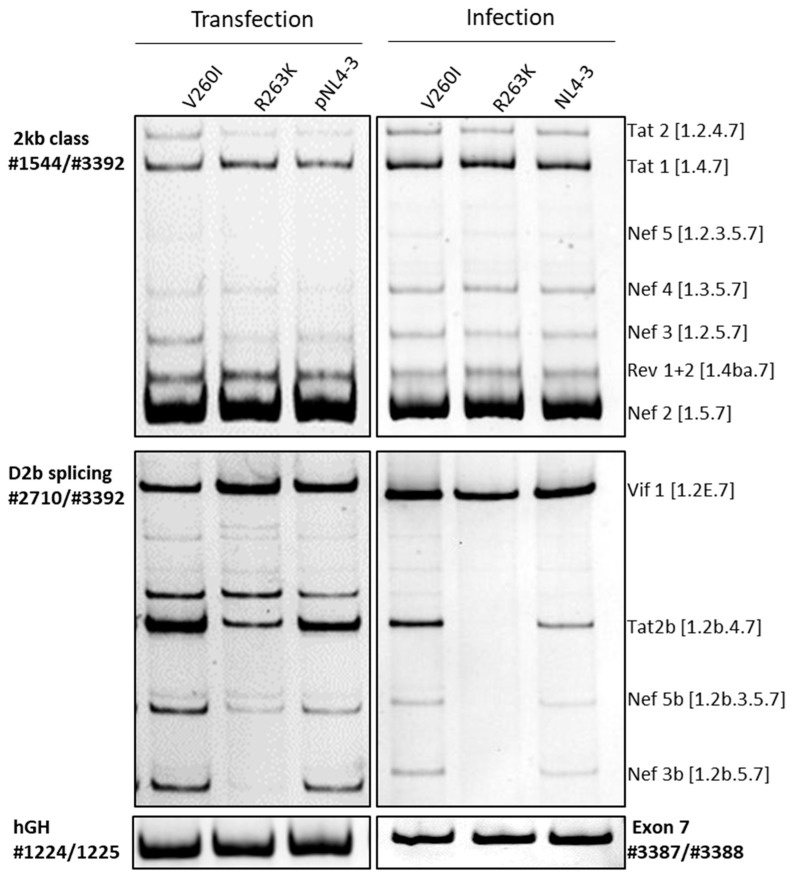
Effects of secondary drug resistance-associated mutations on the HIV-1 splicing pattern. Left panel: Proviral plasmids were used for transfection as wildtype (pNL4-3) or carrying either the V260I or R263K mutation. 2.5 × 10^5^ Hek 293T cells were transiently transfected with 1 μg of each construct together with 1 μg of pXGH5 (hGH) to monitor transfection efficiency. Then, 24 h after transfection, RNA was isolated and subjected to RT-PCR analysis using different primer pairs (#1544/#3392 for 2kb class, #2710/#3392 for D2b splicing, #1224/#1225 for hGH). PCR products were separated by a 10% non-denaturing polyacrylamide gel electrophoresis and stained with ethidium bromide. Right panel: 1.0 × 10^6^ PM1 cells were infected with either wildtype or mutant virus (MOI 0.05). RNA was harvested 72 h post-infection, and RT-PCR was performed with the same primer pairs as for transfection except for the control (#3387/#3388 for Exon 7). Again, PCR products were separated by a 10% non-denaturing polyacrylamide gel electrophoresis and stained with ethidium bromide. The main difference between the splicing patterns can be seen for R263K D2b splicing where the Tat 2b, Nef 5b, and Nef 3b messages are lost upon infection. Experiments were performed as individual triplicates.

Using site-directed mutagenesis, the selected nucleotide exchanges were inserted into the pNL4-3 proviral plasmid coding for an HIV-1 laboratory wild-type strain. This permits the analysis and comparison of the splicing pattern after both transfection and infection. Different splicing patterns were amplified after transfection of HEK293T cells or infection of PM1 cells, which is a commonly used T-cell line in HIV-1 research [36]. Therefore, particular primer sets were used either amplifying transcripts of the 2 kb class or transcripts using splice donors D2 and D2b (#2710/#3632). For monitoring transfection efficiency, a plasmid coding for human growth hormone (hGH) was co-transfected and expressed transcripts amplified using primer pair #1224/#1225. To monitor the infection, a short region of HIV-1 exon 7 was amplified (#3387/#3388). Significant differences were only found in the analysis of the SD2/SD2b splicing pattern, both in comparison with the parental NL4-3 and between the mutants themselves. In the transfection experiments, the V260I variant showed a slight increase in the Tat2b message, which was in contrast to the HEXplorer prediction of a slight decrease in SD2b recognition. A similar pattern could be observed upon infection with the V260I virus (Figure 2). In the transfection experiment, the R263K mutant showed a reduced recognition of splice donor SD2b. This pattern was even more pronounced upon infection of PM1 cells with the R263K virus. Recognition of SD2b was completely abolished, which has a particular effect on the processing of Tat and Nef RNA species (Figure 2). This observation of the full loss of SD2b recognition upon the infection with the R263K virus was confirmed in three independent biological replicates.

**Figure 3:**
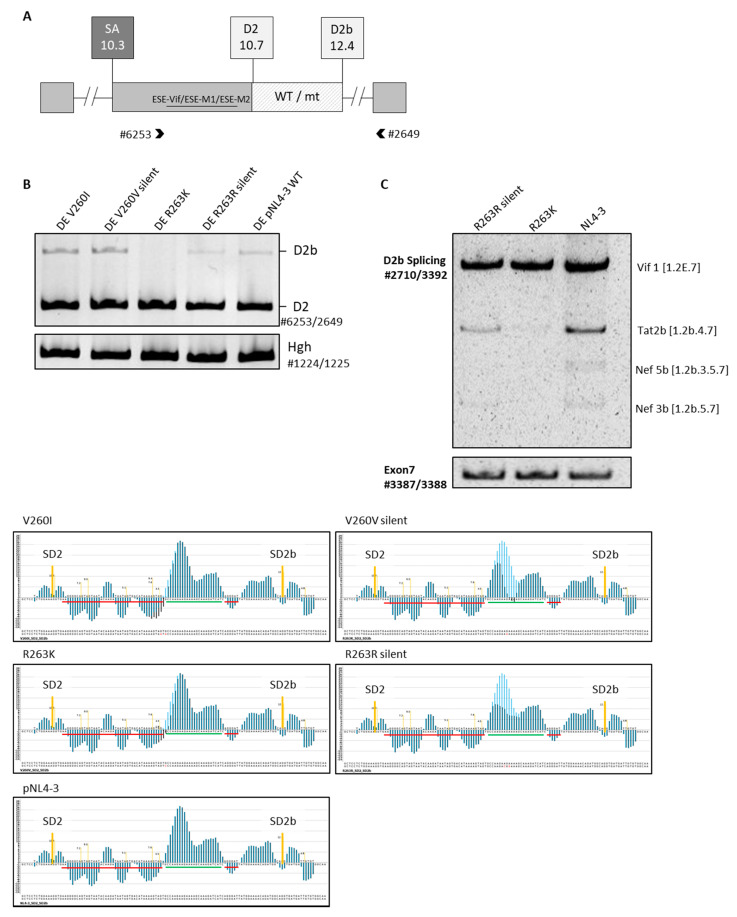
Silent variant of R263K with a comparable effect on SREs: (**A**) schematic overview of the three-exon minigene-splicing reporter containing the HIV-1 native splice donors SD2 and SD2b. Parental or mutated sequences are inserted between the two splice donors. The lower panel shows the respective HEXplorer plots of the inserted sequences, compared with the parental sequence; (**B**) briefly, 2.5 × 10^5^ HeLa cells were transiently transfected with the splicing reporter plasmids, and RNA was harvested 24 h post-transfection. Hgh was co-transfected as a control. RT-PCR samples were run on a non-denaturing 10% polyacrylamide gel; (**C**) HIV-1 proviral plasmids carrying either the wildtype, the missense, or the silent mutation were prepared and used for infection of PM1 cells with an MOI of 0.05. RNA was harvested 72 h post-infection and the splicing pattern was analyzed via RT-PCR on a 10% non-denaturing PAA gel. Experiments were performed as individual triplicates.

To dissect whether the mutations’ effect on replication and splicing is dependent on the altered coding potential of the sequence by the missense mutations, the HEXplorer tool was used to design silent versions of the selected mutations with the same in silico predicted potential to change splicing behavior. For the missense mutation V260I with a ΔHZEI of −37.79, a silent version V260V with a ΔHZEI of −39.0 was generated. For the missense mutation R263K with a ΔHZEI of −132.16, a silent version R263R with a slightly lower ΔHZEI of −107.8 was generated (Figure 3, lower panel). For further analysis, the mutations were also inserted into subgenomic splicing reporters. The three-exon minigene-splicing reporter consists of two exons flanking an amendable middle exon. It carries the naturally occurring splicing regulatory elements of exon 2 as well as the two splice donors SD2 and SD2b. In between the two splice donors, the missense mutations V260I and R263K and the silent mutations V260V and R263R, as well as the parental sequence, were inserted (Figure 3A). Transfection and subsequent RT-PCR analysis of the splicing pattern, in particular recognition of SD2 and SD2b, revealed differences in the splicing pattern between the mutations and compared with the parental NL4-3 sequence. There was no difference in SD2 and SD2b recognition upon insertion of the V260I or V260V silent mutation; however, a slight increase in SD2b recognition could be recognized, compared with the NL4-3 sequence, for both versions of the mutation. For R263K, a similar pattern was observed, as previously seen in the infection experiment (Figure 2) with SD2b recognition fully diminished. Interestingly, the silent version R263R with a similar but even slightly lower ΔHZEI of −107.8 showed only a slight decrease in SD2b recognition, compared with the parental NL4-3 sequence (Figure 3B). These observations were confirmed in three biological replicates.

To further analyze the silent mutation R263R, the mutation was inserted into the pNL4-3 proviral plasmid by site-directed mutagenesis and used for infection. Upon infection of PM1 cells and RNA isolation 72 h post-infection, RT-PCR analysis was carried out using the previously described primers #2710/3392 to analyze the SD2/SD2b usage. As expected from the HEXplorer prediction, the silent R263R mutation led to a similar splicing pattern as R263K, albeit, in contrast to the missense version R263K, a slight band for the most abundant mRNA species Tat2b was still present (Figure 3C). This slight difference in the recognition of SD2b between the silent and missense R263 mutation was confirmed in three independent biological replicates.

## 3. Discussion

By infection and transfection experiments, and the employment of splicing reporters, this work demonstrates that resistance-associated HIV-1 integrase mutations can highly influence the viral splicing pattern.

We report about two secondary resistance-associated mutations, V260I and R263K, located within the C-terminal domain of integrase [30]. Both mutations were selected for further analysis since they are bioinformatically predicted to affect the splicing pattern by disrupting ESE2b and, thus, potentially contribute to an imbalance in HIV-1 mRNA transcripts (Figure 1C).

The SREs mostly affected by these mutations are the recently described ESS2b and ESE2b, which regulate the recognition of splice donors 2 and 2b, as well as the closely located splice acceptor A1 (Figure 1B) [26]. Both splice donors located within leader exon 2 are generally used infrequently; recognition and use of D2, however, exceeds D2b [26,27]. Exon 2/2b recognition particularly contributes to the generation of Vif mRNA species, a counter actor of the host restriction factor APOBEC3G [37], as well as minor Tat mRNA species including Tat2b. Especially the balanced regulation of Vif expression is crucial for viral replication. Recently, Koma et al. described naturally occurring single nucleotide mutations in the terminal *pol* region that are caused by adaptation in an APOBEG restricted setting that led to varied Vif expression levels. They show that excessive or insufficient expression of Vif has detrimental effects on viral replication and suggest that the adaptive mutations alter Vif expression mainly via splicing [34,35]. However, at exon 2, the Vif mRNA overlaps with the integrase reading frame. Therefore, the SREs and potential sequence changes due to mutations in this region can have major impacts on both Vif levels and integrase function. Both contribute greatly to efficient virus replication [38,39].

Transfection and infection experiments revealed that in particular, the R263K mutation, located within ESE2b, contributed to a splicing imbalance where mRNA transcripts employing SD2b are fully diminished (Figure 2). This pattern was reproducible with a silent version of the mutation that had a similar potential to alter ESE2b (Figure 3). R263K was of particular interest since it is described to be the most commonly selected resistance mutation selected under therapy with the second-generation integrase strand-transfer inhibitor (INSTI) dolutegravir (DTG) [40], and it was also recently described to contribute to low-level resistance in patients receiving first-line treatment with DTG [31]. Reports show that R263K reduces DTG susceptibility by approximately twofold [41]. However, the molecular mechanism underlying drug resistance caused by this mutation is yet to be fully understood. A possible explanation of the molecular mechanism of resistance could be an interaction of the non-transferred strand with the transferred viral DNA strand, which results in a change in the positioning of the end of the viral DNA and, thus, could affect the interaction between INSTIs and the DNA [40]. The general effect of the mutation on the viral replication has been described to decrease in integration capacity [32]. Our results reveal that furthermore, the R263K, as well as a silent version of the mutation, can contribute to a highly imbalanced splicing behavior at SD2/SD2b.

The data show that splicing analysis may play an important part in resistance profiling and consequently may contribute to a better understanding of resistance mechanisms regarding integrase mutations. Furthermore, since HIV-1 is generally dependent on a tightly regulated splicing balance and the mRNA isoforms generated, targeting the splicing process has been discussed as a potential approach to disrupting viral replication [42]. One approach along this line is the use of locked nucleic acids (LNA), antisense oligonucleotides forming a particularly stable Watson–Crick base pairing with the RNA through an additional methylene bridge in the ribose sugar. We have recently been able to report that the gymnotic, without transfection reagent, application of LNA mixmers to the cell culture medium of HIV-1 infected cells induces the degradation of viral mRNA carrying the target sequence of the LNAs, and thus inhibit viral replication [43]. Research on the sterile cure of HIV-1 is still ongoing, with one promising example being the use of the Brec1 recombinase that aims to excise the HIV-1 provirus from the host cell genome [44]. While the sterile cure of HIV-1 patients is still extraordinarily rare, it has been achieved previously for very few patients including the two so-called Berlin Patients, one of whom had a preferential genetic background, more precisely the possession of the HLA-B*57 [45], and the other received a stem cell transplant from a CCR5-mutated donor after being diagnosed with acute myeloid leukemia [46]. Another example to this end is the London patient who is still in remission and also received an allogeneic hemopoietic stem cell transplant from a CCR5Δ32/Δ32 donor as the second Berlin patient [47].

The characterization of possible new targets of antiviral agents is an important branch of research, particularly in light of increased drug resistance. In resistance profiling, data presented here imply that mutations changing the splicing outcome can highly influence viral gene expression and might have roles in the emergence of drug resistance.

## 4. Materials and Methods

### 4.1. Proviral Plasmids

Proviral plasmids pNL4-3 V260I, R263K, and R263R were generated by an overlapping-PCR technique (PCR1 with primers a + b, PCR2 with primers c + d, PCR 3 with PCR 1 and 2 as a template using primer a + d) using their respective primer pairs (Table 1).

### 4.2. Three-Exon Minigenes

The three-exon minigenes were derived from the fibrinogen Bß-minigene pT-Bß-IVS7ß 1G > T and were previously described [26]. The middle exon carried HIV-1-derived SD2 and SD2b, as well as splicing regulatory elements ESE-Vif, -M1, and -M2. The respective wildtype and mutated fragments of HIV-1 exon 2/2b were added at their authentic position between SD2 or SD2b, by using primer pairs #5941/#6040 (V260I), #5941/#6042 (V260V silent), #5941/#6041 (R263K) and #5941/#6043 (R263R silent). All oligonucleotides used were obtained from Metabion GmbH.

### 4.3. Expression Plasmids

A plasmid encoding for the human growth hormone hGH (pXGH5) [42] was co-transfected to monitor transfection efficiency.

### 4.4. Cell Culture, Infection, and Transfection

HeLa and HEK293T cells were cultured in Dulbecco’s high-glucose modified Eagle’s medium (DMEM), supplemented with 10% fetal calf serum and 50 g/mL penicillin–streptomycin. PM1 cells were cultured in Roswell Park Memorial Institute Medium (RPMI), supplemented with 10% fetal calf serum and 50 g/mL penicillin–streptomycin. Cells were propagated twice a week. For transient transfection, 2 × 10^5^ cells per well were plated in six-well plates. Transient-transfection experiments were performed using Mirus TransIT-LT1 transfection reagent, according to the manufacturer’s instructions. For infection experiments, PM1 cells were adjusted to 1 × 10^6^ cells per ml and inoculated with virus stock at an MOI of 0.05 for 6 h in 1 mL medium before they were washed with 5ml PBS and kept in 2 mL RPMI at 37 °C.

### 4.5. RNA Isolation and RT-PCR

Either 24 h post-transfection or 72 h post-infection, total cellular RNA was isolated by using acid guanidinium thiocyanate–phenol–chloroform. RNA was reverse transcribed by using Superscript III reverse transcriptase (Invitrogen) and oligo(dT) primers (Invitrogen) for semiquantitative RT-PCR with the denoted primer pairs.

### 4.6. Bioinformatic Tools

The HBond score was calculated via https://www2.hhu.de/rna/html/hbond_score.php (Last accessed on 11 June 2021). The MaxEnt scan algorithm can be accessed via http://hollywood.mit.edu/burgelab/maxent/Xmaxentscan_scoreseq_acc.html (Last accessed on 11 June 2021) and the HEXplorer tool is available under https://www2.hhu.de/rna/html/hexplorer_score.php (Last accessed on 11 June 2021).

## Figures and Tables

**Table 1:** Primer sequences.

RT-PCR:
#1554 CTTGAAAGCGAAAGTAAAGC
#2710 AAGGGGCAGTAGTAATACAA
#3392 CGTCCCAGATAAGTGCTAAGG
#1224 TCTTCCAGCCTCCCATCAGCGTTTGG
#1225 CAACAGAAATCCAACCTAGAGCTGCT
#2649 TCCACCACCGTCTTCTTTAG
#6253 CAACCAAACAACCTTAGGGGA
Cloning:
(a) Proviral plasmids
#3632 (d) TGGATGCTTCCAGGGCTC
#5553 (a) CTGGCAGAAAACAGGGAGATT
V260I as (b) CTTTGCTTTTCTTCTTGGTATTACTTTTATGTCACTATTATC
V260I s (c) GTGACATAAAAGTAATACCAAGAAGAAAAGCAAAGATCATCAG
R263K as (b) GATGATCTTTGCTTTTTTTCTTGGCACTACTTTTATG
R263K s (c) GTAGTGCCAAGAAAAAAAGCAAAGATCATCAGG
R263R s (c) GTAGTGCCAAGACGGAAAGCAAAGATCATCAGG
R263R as (b) GATGATCTTTGCTTTCCGTCTTGGCACTACTTTTATG
(b) Three-exon-minigene
#6040 GGGGCTAGCGCAGTAGTAATACAAGATAATAGTGACATAAAAGTAATACCAAGAAGAAAAGCAAAGATCAT
#6041 GGGGCTAGCGCAGTAGTAATACAAGATAATAGTGACATAAAAGTAGTGCCAAGAAAAAAAGCAAAGATCATCAGGGATTAT
#6042 GGGGCTAGCGCAGTAGTAATACAAGATAATAGTGACATAAAAGTAGTCCCAAGAAGAAAAGCAAAGATCAT
#6043 GGGGCTAGCGCAGTAGTAATACAAGATAATAGTGACATAAAAGTAGTGCCAAGACGGAAAGCAAAGATCATCAGGGATTAT
#5941 GGACAGTGGCTGACAGT

## Data Availability

The data presented in this study are available on request from the corresponding author.
